# Use and Indications of Human Acellular Dermis in Ventral Hernia Repair at a Community Hospital

**DOI:** 10.1155/2012/918345

**Published:** 2012-10-04

**Authors:** William W. Hope, Devan Griner, Ashley Adams, W. Borden Hooks, Thomas V. Clancy

**Affiliations:** South East Area Health Education Center, Department of Surgery, New Hanover Regional Medical Center, P.O. Box 9025, Wilmington, NC 28401, USA

## Abstract

*Background*. To evaluate the use, indications, and short-term outcomes for human acellular dermis. *Methods*. We retrospectively reviewed patients having human acellular dermis placed for ventral hernia repair from January 2008 through October 2009. Demographic information, operative details, and outcomes of patients with and without recurrences were compared; a *P* value <0.05 was considered significant. *Results*. 115 patients met inclusion criteria. The average age was 60 years (range, 24–89). The technique of repair included primary repair with overlay of mesh in 76%, bridge repair in 13%, and underlay in 11%. Average cost of mesh per operation was $3,709 (range $191–10,630). Open repairs were performed in 90% of patients with addition of component separation in 12%. At an average of 13 months, 58 patients were available for followup (50%), with a 47% recurrence rate. The morbidity rate was 48% and the mortality rate was 2%. Technique of repair was the only significant risk factor for recurrence with bridge repairs associated with a higher rate of recurrence (*P* < 0.05). *Conclusions*. The use of biologic grafts for ventral hernia repair is becoming more popular especially in clean cases. Although followup is limited, there remains a high recurrence rate associated with the use of human acellular dermis.

## 1. Introduction

Inguinal and ventral hernia repairs are some of the most common surgical procedures performed worldwide. Good evidence supports the concept of a tension-free repair in most cases with the use of mesh in inguinal and ventral hernia repairs [1–4]. Synthetic meshes, such as polypropylene, polyester, and expanded polytetrafluoroethylene (ePTFE) have been widely used and studied; however, concerns regarding infection have somewhat limited their use and efficacy.

The introduction of biologic grafts in 1999 revolutionized hernia repair and allowed surgeons to place these materials in infected fields. The theoretical advantage of these products for use in infected fields relates to their inherent properties of a regenerative matrix; however, robust scientific evidence supporting this is lacking [5]. Despite this, and largely due to early promising results, biologic grafts have been widely accepted by the surgical community as an alternative to synthetic products in contaminated and potentially infected fields. Increasing interest in the field of biologic mesh has resulted in a dramatic increase in the number and types of biologic products available, with over $400 million spent on biologic grafts in the United States in 2007 [5]. This rapid increase in technology has made choosing the right mesh for each patient quite challenging to the surgeon, and indications for the use of biologic mesh are becoming blurred. Due to this and the paucity of data associated with many of the biologic products, we evaluated our experience with the use of human acellular dermis, one of the most commonly used biologic grafts, its indications, and outcomes for use in ventral hernia repair.

## 2. Methods

Following Institutional Review Board approval, we retrospectively reviewed all patients having human acellular dermis placed for repair of ventral hernias from January 1, 2008 through October 31, 2009. During the study period, there were no limitations on the use of biologic mesh in our hospital. Fifteen general surgeons and one plastic surgeon, with a varying degree of interest and expertise in hernia surgery, performed hernia repairs during the study period. Indications for use of the biologic mesh were left to the discretion of the surgeon. Demographic information, operative details, and outcomes were documented. Follow-up data were obtained from the patients' electronic medical records, and recurrence rates were obtained from physical exam or by imaging, specifically computed tomography (CT) scans. Patients with and without recurrences were compared using *t*-test, Wilcoxon rank sum, chi-squared, and Fisher's exact when appropriate; a *P* value <0.05 was considered significant.

## 3. Results

During the study period, 115 patients met inclusion criteria. The average age was 60 years (range, 24–89 years); 61% were female and 86% Caucasian. Biologic grafts were used in clean hernia repairs in 74% of cases. Alloderm (Life Cell Corp., Branchburg, NJ) was the human acellular dermis used in 79% of cases and FlexHD (Ethicon Inc., Somerville, NJ) was used in the remaining 21%. The average operating room time was 125 minutes (range, 31–420 minutes). Recurrent hernias were present in 18% of patients, with 25-month average time from the previous hernia repair to operation. In the patients with recurrent hernias, 19% had a previous mesh infection. The most common previous operation was open, and the most common mesh used was human acellular dermis (Table 1). A concomitant gastrointestinal or soft tissue surgery was performed in 23% of patients (Table 2). The technique of repair included primary repair with overlay of mesh in 76%, bridge repair in 13%, and underlay in 11%. No patients had repairs with the graft placed in the retromuscular space. The average cost of mesh per operation was $3,709 (range $191–$10,630). Open repairs were performed in 90% of patients with addition of a standard open component separation with incision of the external oblique in 12%. At an average 13 months, 58 patients (50%) were available for followup and had a 47% recurrence rate. The morbidity rate was 48% (Table 3), and the mortality rate was 1.7%. The overall wound infection rate was 14% with 3% of patients having to have their human acellular dermis removed and the remaining 11% able to be treated with antibiotics alone or antibiotics with local wound debridement and graft salvage. The technique of repair was the only significant risk factor for recurrence with bridge repairs associated with a higher rate of recurrence (*P* < 0.05) (Tables 4, 5, 6, and 7). 

## 4. Discussion

Biologic grafts for use in complex hernia repair have been a major breakthrough in the field of hernia surgery. At present, there are 12 different biologic grafts commercially available, which differ based on many unique characteristics including the source material, processing techniques, and handling characteristics. Biologic grafts can initially be divided into heterograft, not from a human source, or allograft, from a cadaveric source.

Human acellular dermis products are widely used and studied biologic products. Several cadaveric allografts are currently available including Alloderm (Life Cell Corporation, Branchburg, NJ), AlloMax (Tutogen Medical Inc, Alachua, FL), and FlexHD (Musculoskeletal Tissue Foundation, Edison, NJ). Alloderm has been the most often used, as it was the first product available, and many series have reported its use for the repair of complex abdominal wall reconstruction. Alloderm is created from cadaveric skin, is noncrosslinked, and freeze dried requiring approximately a 20- to 30-minute soak before use.

Initial reports on the use of Alloderm in complex hernias were promising. Three major centers reported on 171 patients with only a 4% graft removal rate and with an expected high rate of wound complications inherent in this patient population [6–8]. As these studies were preliminary reports, followup was short and difficult to interpret as were the techniques of repair. Further studies from several authors reported a laxity associated with the use of Alloderm, especially when this material was used as a “bridge” or interposition repair. Blatnik and colleagues reported on 11 patients who underwent complex abdominal hernia repairs and reported 80% recurrence rate at a mean followup of 24 months [9]. Candage and colleagues reported on a larger series with a subset of 20 patients that underwent a bridge repair [10]. They reported a 30% recurrence rate with 88% of the patients who developed eventration having a “bridge” repair. Bluebon-Langer and colleagues reported on 7 patients who underwent component separation with an interposition repair with Alloderm, observing the laxity within 12 months [11]. Jin and colleagues also reported on the effect that technique of repair has on outcomes in 37 patients undergoing abdominal wall repair with Alloderm showing that it should be used only as reinforcement after primary fascial reapproximation [12]. Based on these reports, it was evident that technique of repair had an important impact on the recurrence rate associated with these biologic materials. Our findings echo these reports in that the technique of repair was the only significant risk factor for recurrence. The “bridge” repairs had a significantly higher recurrence rate than onlay or underlay techniques. While the high recurrence rate associated with bridging repairs is well known today and has been previously reported [12], most of our bridging repairs were performed early in our experience and show the unrealized high expectations that these surgeons had for these new biologic products. In our bridging repairs only 1 patient had it placed as a temporary abdominal closure technique. 

Our series is unique to previously published reports in many ways. First, our report is from a community teaching hospital comprised of private practitioners and academic surgeons differing from many previous reports from large trauma centers or specialized hernia centers that have a specific focus on abdominal wall reconstruction. Second, a surprising finding of our study is that the majority of cases in which the biologic graft was used were clean cases, whereas the majority of prior studies have been in complex or infected hernia repairs, because this was the accepted initial indication for these products. Interestingly, we report 6 cases where human acellular dermis was used in a recurrent hernia where the previous mesh was human acellular dermis. In review of these cases, 5 of the cases were clean with one being contaminated. Only one of the cases had a different surgeon performing the first and second operation and in this case case the first surgeon used Alloderm and the second surgeon used FlexHD. The rationale for repeat use of the biologic is unknown and difficult to explain with 3 of these 6 patients having a documented repeat recurrence. Our report shows that with increased experience with these products, surgeons are liberalizing the indications for the use despite little supporting scientific literature. 

One potential drawback of these biologic products is the cost. Several studies have evaluated the cost associated with the use of these products recommending caution in their use until further studies and information are available [5, 9]. Several hospitals have also limited the use of these products to “hernia specialist” or for complex/infected cases. During this study, our hospital had no constraints on the use of biologic products; however, it had limitations relating to the types of biologic products available. One impetus for this study was to evaluate our use of these products and the economic impact for our hospital. Although no direct comparisons were made, the cost of biologic mesh for a clean case far exceeds that for a synthetic mesh at our hospital; however, we cannot comment further on the economic impact as we did not evaluate hernia repairs using synthetic mesh. Cost comparisons are also very difficult due to the followup needed to evaluate for long-term complications such as mesh infections and recurrences. 

There are several limitations to our study. One is that the indication for use of biologic mesh at our community hospital was surgeon dependent and this has caused liberalization of use that may not be seen in other hospitals. It is evident that our surgeons incorrectly bought into the premise that these human acellular grafts would be as strong as synthetic mesh and could be used in clean and infected fields without the possibility of infections. Secondly, our followup is fairly limited due to the nature of multiple different surgeons, from multiple different groups, with different followup patterns performing these operations and this could affect our recurrence rate. Although our followup is limited, our series reports a high recurrence rate, especially for a series with many clean cases and likely with improved followup our recurrence rate may be even higher. One caveat to our recurrence rate is that recurrences were often found on CT scans obtained for indications other than to evaluate for recurrent hernias, and many of these recurrences were asymptomatic, so patients did not undergo further surgery. Despite this, based on our surgeons' experience and appreciation of this high recurrence rate, many surgeons in our hospital have begun to use a porcine dermis product for ventral hernia repairs, as it is believed to result in less laxity and stretching. Technique of repair has also been discussed with a near abandonment of the “bridge” repair except in extremely complex cases where temporary closure is needed and increasing use of component separation. 

In conclusion, we report on the use of acellular human dermis for ventral hernia repair including results from many surgeons using different techniques with a high rate of usage in clean cases. Our results show a high recurrence rate associated with their use and that the technique of repair significantly impacts recurrence rates with the interposition or “bridge” repairs resulting in a significantly higher rate compared with an underlay or overlay technique. Based on our surgeons experiences the use of human acellular dermis in clean ventral hernia repairs should be abandoned secondary to the unacceptable high recurrence rate and increased cost. Secondary to our results, our hospital has shifted to using different biologic products for ventral hernia repair and have put more emphasis on outcomes associated with these products.

## Figures and Tables

**Table 1:** Characteristics of the 26 patients who had a recurrent hernia operation with the use of acellular human dermis.

Type of most recent operation	
Open	76%
Lap	19%
Unknown	5%

Previous mesh	

Human acellular dermis	24%
ePTFE	19%
Composite, bard/ventralex	14%
Proceed surgical mesh	5%
Polypropylene	10%
Suture	14%
Unknown	14%

ePTFE: expanded polytetrafluoroethylene.

**Table 2:** Type of concomitant surgery performed in patients undergoing hernia repair.

Associated cases	
Colostomy/ileostomy takedown	3%
Small bowel resection	8%
Nissen/foregut surgery	3%
Colectomy	6%
Above knee amputation	1%
Cholecystectomy	1%
Cystorrhaphy	1%
None	77%

**Table 3:** Type and percentage of morbidity following hernia repair.

Morbidity	Percentage
None	52%
Ileus	20%
Urinary retention	2%
Anemia	3%
Atelectasis	3%
Respiratory failure/insufficiency	7%
Renal failure/insufficiency	2%
Atrial fibrillation	2%
Altered mental status/confusion	2%
Wound infection	11%
Infection requiring mesh removal	3%

**Table 4:** Comparison of recurrence rates based on demographics and patient factors.

Variable	Recurrent	Not recurrent	*P* value
Age (years)	62.8 ± 13.9	54.7 ± 15.7	0.5
Gender			
Male	38%	62%	0.3
Female	52%	48%	
Race			
White	46%	50%	1.0
Black	54%	50%	
Incarcerated	30%	10%	0.053
Type of hernia			
Incisional	85%	84%	0.53
Ventral	15%	10%	
Parastomal	0%	6%	
ASA			
1	0%	3%	0.9
2	30%	23%	
3	55%	61%	
4	15%	10%	
5	0%	3%	

**Table 5:** Comparisons of recurrence rate based on previous hernia surgery.

Variable	Recurrence	No recurrence	*P* value
Recurrent	26%	23%	0.8

Of recurrent cases:			

Previous mesh infection	29%	14%	1.0
Type of most recent operation			
Open	71%	29%	1.0
Lap	71%	29%	
Previous mesh			
Human acellular dermis	43%	14%	0.4
ePTFE	43%	14%	
PTFE/polypropylene	14%	14%	
Polypropylene	0%	30%	
Suture	0%	14%	
Unknown	0%	14%	

ePTFE: polytetrafluoroethylene.

**Table 6:** Comparisons of recurrence rates based on class of case and thickness of graft.

Variables	Recurrence	No recurrence	*P* value
Class			
Clean	78%	71%	0.4
Clean-contaminated	22%	19%	
Contaminated	0%	10%	
Removal Mesh/Infected	0%	6%	0.5
Thickness			
Normal (<0.8 mm)	81%	61%	0.06
Intermediate (0.8–1.7 mm)	4%	0%	
Ultrathick (>1.8 mm)	15%	39%	

**Table 7:** Comparisons of recurrence rate based on operative details.

Variables	Recurrence	No recurrence	*P* value
Associated cases	22%	19%	0.8
Method of procedure			
Open	78%	87%	0.4
Laparoscopic	11%	0%	
Lap converted to open	11%	13%	
Component separation	11%	16%	0.7
Type of repair			
Primary closure w/overlay	52%	81%	
Bridge	30%	6%	**0.040**
Underlay	18%	13%	
Amount of mesh (cm^2^)	108.3 ± 94.5	114.9 ± 94.5	0.6
OR time (minutes)	152.9 ± 99.7	119.4 ± 88.6	0.1
Estimated blood loss (mL)	71.85 ± 85.1	97.3 ± 155.8	1.0
